# Do breast cancer survivors with a recent history of clinical depression report worse experiences with care? A retrospective cohort study using SEER‐CAHPS data

**DOI:** 10.1002/cam4.5031

**Published:** 2022-08-05

**Authors:** Mariana Arevalo, Trevor A. Pickering, Sally W. Vernon, Kayo Fujimoto, Melissa F. Peskin, Albert J. Farias

**Affiliations:** ^1^ Department of Health Promotion & Behavioral Sciences School of Public Health, The University of Texas Health Science Center at Houston (UTHealth) Houston Texas USA; ^2^ Department of Preventive Medicine University of Southern California Los Angeles California USA; ^3^ Department of Preventive Medicine Keck School of Medicine of the University of Southern California Los Angeles California USA; ^4^ Gehr Family Center for Health System Science Keck School of Medicine of the University of Southern California Los Angeles California USA; ^5^ Cancer Control Research Program University of Southern California Norris Comprehensive Cancer Center Los Angeles California USA

**Keywords:** CAHPS, depression, ethnic groups, healthcare disparities, Medicare, mental health

## Abstract

**Purpose:**

We examined whether breast cancer survivors' experiences with care differed by a recent history of clinical depression, and whether associations differed by race/ethnicity.

**Methods:**

Using the Epidemiology and End Results‐Consumer Assessment of Healthcare Providers and Systems (SEER‐CAHPS) dataset, we analyzed records of breast cancer survivors who completed a survey at least 12 months after their cancer diagnosis. We assessed clinical depression 12 months prior to survey completion using Medicare claims. We used separate multivariable logistic regressions to examine the associations between depression and excellent (vs. less than excellent) ratings of experiences with care (i.e., doctor communication, getting needed care, getting care quickly, getting prescription drugs, specialist and overall care). We also assessed interactions of depression by race/ethnicity. All models were adjusted for demographics and cancer prognostic and treatment factors.

**Results:**

Of the 2271 survivors, 7.6% were clinically depressed. After adjusting for covariates, survivors with clinical depression had lower odds of reporting excellent ratings of their ability to get needed care, care by their specialist, and overall care, compared to those without depression (AOR = 0.58, 95% CI: 0.40–0.84; AOR = 0.40, CI: 0.31–0.76; and AOR = 0.61, CI: 0.42–0.89, respectively). Among Hispanics, having depression was associated with higher odds of excellent ratings of one's ability to get needed care (AOR: 5.42, 95% CI: 1.02–28.81). No other statistically significant associations by race/ethnicity were found.

**Conclusions:**

Breast cancer survivors with depression report poorer patient experiences with care. Further research is needed to understand complexities of ratings of experiences with care among survivors of diverse backgrounds.

**Implications:**

Survivors with a recent history of clinical depression may benefit from additional supportive care services.

## Graphical abstract

Breast cancer survivors with a recent history of clinical depression were less likely to report excellent ratings of care on three domains assessing experiences of care, compared to those without a recent history of depression. Survivors with a recent history of depression appear to be particularly vulnerable to difficulties in some of their healthcare encounters, as reflected by their reported ratings of care.

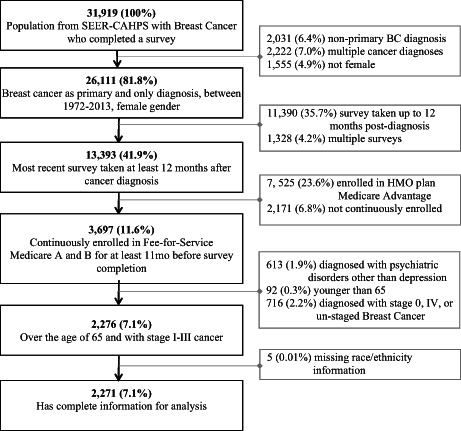

## 1 BACKGROUND

Quality patient‐centered care is multi‐faceted, but patients' experiences of care are at the cornerstone of this concept.^1^ Key aspects of care are frequently measured through reports of patient experiences during healthcare encounters such as perceptions about ease, access, and coordination of care.^1^ Research on patient experiences with care is important for assessing continuity of care, strong patient‐provider relationships, and improved outcomes.^2^ Positive patient experiences with care have been linked to increased treatment adherence, better clinical outcomes, and reduced unnecessary healthcare utilization.^3^ Researchers have studied the association between patient experiences with care and patient demographic characteristics,^4^, ^5^, ^6^, ^7^ but little research has focused on examining the impact of clinical depression on patient experiences with care. Individuals with depression are less likely to receive standard levels of care^8^ and more likely to have worse cancer outcomes.^9^, ^10^, ^11^, ^12^

Depression is a common psychiatric comorbidity after cancer.^13^ Stressful times after a breast cancer diagnosis and treatment often lead to psychological distress, fatigue, trouble sleeping, anhedonia, among other symptoms.^14^ These symptoms can also turn into clinically significant levels of depression.^14^ Patients with depression may experience increase perceptivity of social interactions,^15^ possess different motivations and abilities to navigate healthcare encounters, and this may result in varying experiences with care.^16^ Prior research shows that individuals with depression symptoms report worse ratings of experiences with care, such as more difficulty getting care quickly, getting needed care, communicating with their doctor, and less favorable ratings of care from their personal doctor, specialist, and overall care, compared with individuals without depressive symptoms.^17^ However, prior studies examined individuals who were cancer‐free and had self‐reported depressive symptoms.^16^, ^17^ To our knowledge, experiences with care have not been examined among breast cancer survivors with clinically diagnosed depression.

With an increased emphasis to improve quality care for underserved cancer survivor populations,^18^ it is important not only to examine whether mental health has a differential impact on experiences with care, but also examine differences by race/ethnicity. Racial/ethnic minorities are disproportionally affected by breast cancer,^19^ but there is a documented lack of targeted cancer care for racial/ethnic minorities.^20^ Prior research shows that minority breast cancer survivors report worse ratings of their ability to get care quickly, get needed care, get needed prescriptions, and ratings of their overall care and care by their specialists, compared to non‐Hispanic White cancer patients.^4^, ^21^ Yet, little is known about care experiences of racial/ethnic minority cancer survivors with clinically diagnosed depression.

Therefore, the primary objective of our study was to determine whether patient experiences with care differed among breast cancer patients with and without a recent history of clinical depression. We hypothesized that breast cancer survivors with a recent history of depression would report poorer ratings of care compared to those without a history of depression. A secondary objective was to determine whether clinical depression was more detrimental on patient experiences with care depending on the respondents' race/ethnicity. We hypothesized that racial/ethnic minorities with depression would report worse experiences with care, compared to Whites with depression.

## 2 METHODS

**FIGURE 1:**
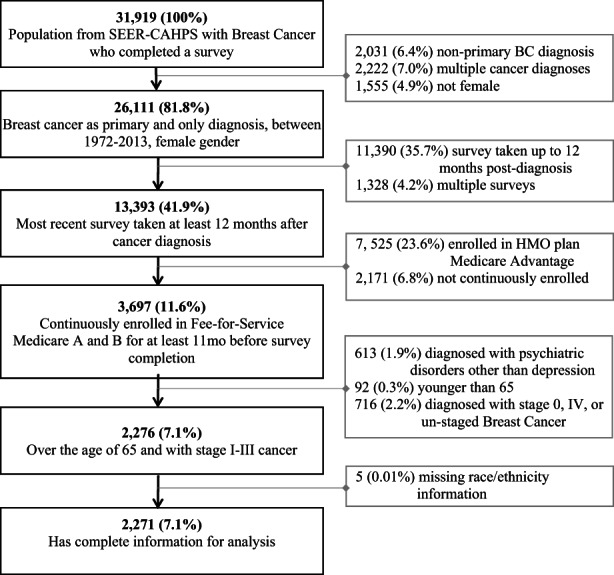
CONSORT diagram for study population

We conducted a retrospective cohort study using the Epidemiology and End Results‐Consumer Assessment of Healthcare Providers and Systems (SEER‐CAHPS) linked dataset.^22^ We analyzed records of women aged 65 and older, who received a primary (and only) diagnosis of non‐metastatic, invasive breast cancer between the years 2000 and 2013, who completed a CAHPS survey at least 12 months after their cancer diagnosis, and who were continuously enrolled in fee‐for‐service Medicare part A and B. Continuous enrollment was considered as enrollment on the fifteenth day of the month for the 12 consecutive months prior to survey completion, with no more than a 1‐month interruption. It is possible for individuals to complete more than one CAHPS survey. Therefore, for individuals with multiple eligible CAHPS surveys, we selected the CAHPS survey completed closest to the date after their cancer diagnosis. We excluded individuals younger than 65 years of age, enrolled in managed care or Health Maintenance Organization (HMO) plans, diagnosed with psychiatric disorders other than depression, and whose race/ethnicity information was missing (Figure 1). Individuals younger than 65 were excluded because in order to qualify for Medicare they must have end‐stage renal disease, or amyotrophic lateral sclerosis, among other criteria, and this population may have unique health needs that should be examined independently.^23^ Individuals enrolled in managed care or HMOs were excluded because we did not have access to their medical claims. Lastly, we did not include individuals with psychiatric diagnosis other than depression because we were interested in the relation between clinical depression and patient experiences with care. This study was reviewed and granted exempt status by the UTHealth Committee for Protection of Human Subjects (HSC‐SPH‐20‐0812).

### 2.1 Study variables

The outcome variables, patient experiences with care, were assessed using two global ratings and four composite reports. We selected these CAHPS measures because previous research found that race/ethnicity and depression status varied by these characteristics.^4^, ^17^, ^21^ The two global measures selected were single‐item ratings of *Overall Care*, and *Care by Specialist*, scored on a 0‐to‐10 scale, with higher scores indicating better ratings of care.^24^ The four selected composite measures were multi‐item measures that assessed the following domains: *Doctor Communication*, *Getting Care Quickly*, *Getting Needed Care*, *and Getting Needed Prescriptions*. Survey respondents were asked to answer each item thinking of their experiences with care in the last 6 months using the following response scale: 1 = never, 2 = sometimes, 3 = usually, and 4 = always. The original response scales for the composite measures were transformed to a 0‐to‐100 scale using linear scoring,^25^ where higher scores indicated better reports of care. Lastly, patient experiences with care scores (i.e., composite reports and global ratings) were later dichotomized for analysis, as described in the analysis section. More information about CAHPS measures can be obtained elsewhere.^26^

The independent variable was clinical depression status. We identified breast cancer patients with at least two distinct medical claims with a diagnostic code for depression at least 12 months before survey completion. We used the International Classification of Disease, 9th edition (ICD‐9) and Current Procedural Terminology codes to identify individuals clinically diagnosed with depression (Supplemental Appendix, Table A1). The study consisted of two groups: (i) a group comprised of individuals clinically diagnosed with depression (as defined above), and (ii) a comparison group of individuals with no recent history of depression or any psychiatric diagnosis. To form the comparison group, individuals diagnosed with any psychiatric disorder were identified using ICD‐9 codes: 290–319, and removed from the group.

We included the following covariates based on prior research^4^: race and ethnicity (Non‐Hispanic White, Non‐Hispanic Black, Hispanic, Asian), education, age at the time of the completion of the CAHPS survey, time since survey completion, Medicare plan, geographic region, and marital status. Cancer prognosis and treatment covariates included: disease stage, number of self‐reported comorbidities, surgery treatment, radiation treatment. Survey administration mode and year of survey completion were also adjusted for in the models. We controlled for time from diagnosis to survey completion to account for changes over time.

### 2.2 Statistical analysis

Pearson chi‐square tests were conducted to assess participant demographic and clinical characteristics of those with depression versus those with no depression. Because respondents tended to rate their experiences with care very highly, our data were negatively skewed. For this reason, we categorized the patient experiences scores into binary variables, as follows: 0 = scores of less than 90, and 1 = scores of 90 to 100, indicating less than excellent ratings and excellent ratings of experiences with care, respectively, similar to other researchers who have also dichotomized CAHPS measure scores.^6^, ^27^ The analytic sample for each outcome varied because response rates for each item depended on the survivors' contact with the healthcare system, whether certain items were included the year they took the survey, possible participant non‐response, and other intended and unintended missingness.^28^ To examine the association between patient‐reported experiences with care and depression, we used separate multivariable logistic regression models for each global rating and composite measure, adjusted for demographic and clinical covariates. In order to account for the time between cancer diagnosis and survey completion, we adjusted for time since survey completion in all multivariable models. We assessed interactions between race/ethnicity categories and depression status for all models. We conducted stratified analyses when results indicated that there were significant interactions. Statistical significance was established at alpha <0.05. Significance tests and confidence intervals for the estimates were 2‐sided. All analyses were conducted in Stata statistical software version 16.^29^

## 3 RESULTS

Our overall study sample consisted of 2271 breast cancer survivors (Figure 1).

We classified 172 (7.6%) individuals as having a recent diagnosis of clinical depression. The comparison group consisted of 2099 (92.4%) individuals without a recent diagnosis of depression or any other psychiatric diagnosis. As shown in Table 1, a higher percentage of survivors with depression reported two or more comorbidities, compared to those without depression (18.6% vs. 10%, respectively), and reported completing the survey by mail at a higher percentage compared to those without depression (26.2% vs 14.5%, respectively). We found significant mean score differences in ratings of patients' ability to get needed care, get needed prescriptions, and their ratings of their specialist care, and overall care. For example, patients with depression reported significantly lower mean scores in their ability to get needed care, compared to patients without depression (84.4 vs. 89.6, *p* = 0.001; Table 1). Compared to White survivors, a significantly higher proportion of minority survivors reported more than one comorbidity, regional stage cancer II and III, and enrollment in Fee‐for‐Service (FFS) Only plans; except for Asian Americans (Table 2).

**TABLE 1:** Sample characteristics

	Total (*N* = 2271)	No depression (*n* = 2099)	Depression (*n* = 172)	*p* value*
	*n* (%)	*n* (%)	*n* (%)	
Age at survey				
Mean (SD)	79.5 (6.5)	79.5 (6.4)	79.4 (7.1)	0.812
65–70	144 (6.3)	124 (5.9)	20 (11.6)	0.010
71–75	451 (19.9)	421 (20.1)	30 (17.5)	
76–80	671 (29.6)	625 (29.8)	46 (26.7)	
81–85	534 (23.5)	502 (23.9)	32 (18.6)	
86+	471 (20.7)	427 (20.3)	44 (25.6)	
Race/Ethnicity				0.377
Non‐Hispanic White	1903 (83.9)	1762 (83.9)	144 (83.7)	
Non‐Hispanic Black	123 (5.7)	<6%	<7%	
Hispanic	134 (5.9)	121 (5.8)	13 (7.6)	
Asian	102 (4.5)	<5%	<7%	
Marital status				0.298
Not married	1030 (45.4)	943 (44.9)	87 (50.6)	
Married	1174 (51.7)	<52%	<48%	
Missing	67 (2.9)	<4%	<7%	
Education level				0.399
HS or less	1154 (50.8)	1058 (50.4)	96 (55.8)	
Some college+	1056 (46.5)	<47%	<42%	
Missing	61 (2.7)	<3%	<7%	
Self‐reported comorbidities				0.001
0	1504 (66.2)	1407 (67.0)	97 (56.4)	
1	526 (23.2)	483 (23.0)	43 (25.0)	
2+	241 (10.6)	209 (10.0)	32 (18.6)	
Tumor stage				0.053
I	1449 (63.8)	1351 (64.4)	98 (56.9)	
II–III	822 (36.2)	748 (35.6)	74 (43.1)	
Medicare status				0.481
FFS Only	985 (43.4)	906 (43.2)	79 (45.9)	
FFS PDP	1286 (56.6)	1193 (56.2)	93 (54.1)	
Surgery				0.242
No	<1%	<1%	<7%	
Yes	2247 (98.9)	2078 (99.0)	169 (98.3)	
Unknown	<1%	<1%	<7%	
Radiation treatment				0.073
No	1118 (49.2)	1020 (48.6)	98 (57.0)	
Yes	1131 (49.8)	<51%	<42%	
Missing	22 (0.9)	<2%	<7%	
Region				0.804
West	1053 (46.4)	978 (46.6)	75 (43.6)	
Midwest	296 (13.0)	270 (12.9)	26 (15.1)	
Northeast	452 (19.9)	418 (19.9)	34 (19.8)	
South	470 (20.7)	433 (20.6)	37 (21.5)	
Survey administration mode				<0.001
Mail	349 (15.4)	304 (14.5)	45 (26.2)	
Phone	1922 (84.6)	1795 (85.5)	127 (73.8)	
Survey year				0.097
2000–2004	307 (13.5)	293 (13.9)	14 (8.1)	
2007–2010	1064 (46.9)	977 (46.5)	87 (50.6)	
2011–2013	900 (39.6)	829 (39.6)	71 (41.3)	
Time from diagnosis to survey completion				0.292
1–≤2 years	420 (18.5)	392 (18.7)	28 (16.3)	
>2–5 years	954 (42.0)	884 (42.1)	70 (40.7)	
>5–10 years	753 (33.2)	<33%	<39%	
>10–15 years	144 (6.3)	<7%	<7%	
Patient experiences	Mean (SD)	Mean (SD)	Mean (SD)	
Doctor communication	89.8 (15.9)	89.9 (15.7)	88.1 (19.6)	0.188
Getting care quickly	84.0 (23.4)	84.1 (23.4)	82.0 (23.3)	0.305
Getting needed care	89.2 (17.7)	89.6 (17.3)	84.4 (21.6)	0.001
Getting needed prescriptions	92.1 (17.6)	92.3 (17.3)	89.4 (20.3)	0.042
Care by specialist	90.9 (14.3)	91.2 (14.0)	87.0 (17.0)	0.004
Overall care	88.2 (15.6)	88.4 (15.6)	86.5 (14.6)	0.186

* *p*‐value represents two‐sided *t*‐test, Chi‐square test, or Fisher's exact test.

Abbreviations: FFS, fee for service; PDP, prescription drug plan.

**TABLE 2:** Sample characteristics by race/ethnicity

	Non‐Hispanic White (*n* = 1906)	Non‐Hispanic Black (*n* = 129)	Hispanic (*n* = 134)	Asian (*n* = 102)	*p*‐value
	*n* (%)	*n* (%)	*n* (%)	*n* (%)	
Age at survey					
Mean (SD)	79.6 (6.5)	77.2 (6.1)	<7%	<7%	0.004
65–70	115 (6.0)	14 (10.8)	<9%	<11%	0.048
71–75	368 (19.3)	36 (27.9)	26 (19.4)	21 (20.6)	
76–80	566 (29.7)	32 (24.8)	40 (29.9)	33 (32.4)	
81–85	443 (23.2)	34 (26.4)	31 (23.1)	26 (25.5)	
86+	414 (21.8)	13 (10.1)	26 (19.4)	18 (17.6)	
Marital status					<0.000
Not married	837 (43.9)	86 (66.7)	72 (53.7)	35 (34.3)	
Married	1009 (52.9)	<29%	<46%	67 (65.7)	
Missing	60 (3.2)	<9%	<9%	0 (0.0)	
Education level					<0.000
HS or less	929 (48.7)	94 (72.9)	86 (64.2)	45 (44.1)	
Some college+	930 (48.8)	<24%	<33%	<52%	
Missing	47 (2.5)	<9%	<9%	<11%	
Number of self‐reported comorbidities					<0.000
0	1300 (68.2)	74 (57.4)	71 (53.0)	59 (57.9)	
1	412 (21.6)	35 (27.1)	<34%	<34%	
2+	194 (10.2)	20 (15.5)	<14%	<11%	
Tumor stage					0.008
I	1242 (65.2)	71 (55.0)	72 (53.7)	64 (62.8)	
II–III	664 (34.8)	58 (45.0)	62 (46.3)	38 (37.2)	
Medicare status					0.081
FFS PDP	812 (42.6)	65 (50.4)	68 (50.7)	40 (39.2)	
FFS Only	1094 (57.4)	64 (49.6)	66 (49.3)	62 (60.8)	
Surgery					0.727
No	<1%	<9%	<9%	0 (0.0)	
Yes	1885 (98.9)	127 (98.4)	133 (99.3)	102 (100)	
Missing	<1%	<9%	<1%	0 (0.0)	
Radiation treatment					0.438
No	941 (49.4)	71 (55.0)	62 (46.3)	44 (43.1)	
Yes	<50%	<45%	72 (53.7)	58 (56.9)	
Missing	<11%	<9%	0 (0.0)	0 (0.0)	
Region					<0.000
West	833 (43.7)	24 (18.6)	100 (74.6)	96 (94.2)	
Midwest	281 (14.7)	15 (11.6)	0 (0.0)	0 (0.0)	
Northeast	392 (20.6)	34 (26.4)	<18%	<11%	
South	400 (21.0)	56 (43.4)	<9%	<11%	
Survey administration mode					<0.0000
Mail	275 (14.4)	37 (28.7)	29 (21.6)	8 (7.8)	
Phone	1631 (85.6)	92 (71.3)	105 (78.4)	94 (92.2)	
Survey year					0.110
2000–2004	268 (14.1)	14 (10.9)	<12%	<11%	
2007–2010	881 (46.2)	68 (52.7)	<55%	<42%	
2011–2013	757 (39.7)	47 (36.4)	45 (33.6)	51 (50.0)	
Time from diagnosis to survey completion					0.718
1–≤2 years	354 (18.6)	23 (17.8)	27 (20.1)	16 (15.7)	
>2–5 years	789 (41.4)	64 (49.6)	60 (44.8)	41 (40.2)	
>5–10 years	639 (33.5)	<28%	<30%	<39%	
>10–15 years	124 (6.5)	<9%	<9%	<11%	
Patient experiences					
Doctor Communication	89.9	89.3	89.2	89.1	0.935
Getting care quickly	84.4	83.3	81.2	80.4	0.301
Getting needed care	89.3	90.8	87.7	86.9	0.467
Getting needed prescriptions	92.3	89.4	90.4	93.3	0.210
Care by specialist	90.9	88.5	91.5	91.3	0.601
Overall care	88.5	85.6	87.9	86.6	0.266

*Note*: *p*‐value represents two‐sided *t*‐test, chi‐square test, Fisher's exact test, or one‐way ANOVA test.

### 3.1 Effects of depression status on patient experiences with care

Compared to survivors who did not have a recent history of depression, those with depression had lower odds of having excellent reports of their ability to get needed care (aOR:0.58, 95% CI: 0.40–0.83), lower odds of having excellent ratings of care by a specialist (aOR: 0.51, 95% CI: 0.33–0.78), and lower odds of having excellent ratings of overall care (aOR: 0.61, 95% CI: 0.42–0.87; Table 3, Model 1). This relation persisted even after adjusting for demographics and clinical characteristics; survivors who had a recent history of depression had lower odds of having excellent reports of their ability to get needed care (aOR: 0.58, 95% CI: 0.40–0.84), lower odds of ratings of care by a specialist (aOR: 0.51, 95% CI: 0.33–0.78), and lower odds of ratings of overall care (aOR: 0.61, 95% CI: 0.42–0.87), compared to those without a recent history of depression (Table 3, Model 3). Complete results for Model 3 are shown in the Table A1.

**TABLE 3:** Results from logistic regression models assessing the association between depression and experiences with care, unadjusted and adjusted for demographic and clinical characteristics

Measure of patient experience	Doctor communication (*n* = 1698)	Getting Care quickly (*n* = 1747)	Getting Needed Care (*n* = 1660)	Getting Prescription Drugs (*n* = 2028)	Care by Specialist (*n* = 1332)	Overall Care (*n* = 1785)
	OR [95%CI] *p*‐value	OR [95%CI] *p*‐value	OR [95%CI] *p*‐value	OR [95%CI] *p*‐value	OR [95%CI] *p*‐value	OR [95%CI] *p*‐value
Model 1: Unadjusted	0.92 [0.63–1.36] 0.701	0.75 [0.53–1.07] 0.115	0.58** [0.40–0.83] 0.003	0.71 [0.49–1.02] 0.68	0.51** [0.33–0.78] 0.002	0.61** [0.42–0.87] 0.007
Model 2: Adjusted for demographics	0.88 [0.60–1.30] 0.519	0.76 [0.53–1.08] 0.125	0.57** [0.40–0.83] 0.004	0.73 [0.50–1.07] 0.109	0.48*** [0.31–0.74] 0.001	0.61** [0.42–0.88] 0.009
Model 3: Adjusted for demographics and clinical characteristics	0.89 [0.60–1.31] 0.556	0.77 [0.54–1.10] 0.161	0.58** [0.40–0.84] 0.004	0.74 [0.50–1.07] 0.115	0.49*** [0.31–0.76] 0.001	0.61** [0.42–0.89] 0.009

*Note*: *p < 0.05, **p < 0.01, ***p < 0.001. Model 2 was adjusted for age at survey, race/ethnicity, marital status, educational level, Medicare plan, region, survey administration mode, survey year, and time from diagnosis to survey completion (continuous). Model 3 was adjusted for all variables in model 2 and number of self‐reported comorbidities, tumor stage, receipt of surgery, and receipt of radiation therapy.

### 3.2 Patient experiences with care by depression status and race/ethnicity

Among Hispanics, depression was associated with higher odds of excellent reports of their ability to get needed care (aOR: 5.42, 95% CI: 1.02–28.81; Table 4). Among Blacks, depression was non‐significantly associated with higher odds of excellent reports of their ability to get needed care (aOR: 2.51, 95% CI: 0.43–14.79). Among Asians, depression was non‐significantly associated with lower odds of excellent reports of getting needed care (aOR: 0.95, 95% CI: 0.08–11.65). We conducted a stratified model assessing depression status on reports of patients' ability to get needed care among Hispanics (*n* = 95), but the sample size was too small to detect a statistically significant association between depression status and reports of their ability to get needed care (data not shown – see appendices, Table A2). We did not find interactions between depression status and race/ethnic background in any other group for all the other patient experiences with care.

**TABLE 4:** Results from logistic regression models assessing interaction effects race/ethnicity by depression status

	Doctor Communication			Getting Care Quickly			Getting Needed Care			Getting Prescription Drugs			Care by Specialist			Overall Care		
	OR	95%	CI	OR	95%	CI	OR	95%	CI	OR	95%	CI	OR	95%	CI	OR	95%	CI
*N* analyzed	1698			1747			1660			2028			1332			1783		
Depression status																		
No	Ref			Ref			Ref			Ref			Ref			Ref		
Yes	0.87	0.57	1.33	0.68	0.46	1.01	0.48***	0.32	0.73	0.73	0.48	1.10	0.45**	0.28	0.72	0.58**	0.39	0.87
Race/Ethnicity																		
Non‐Hispanic White	Ref			Ref			Ref			Ref			Ref			Ref		
Non‐Hispanic Black	0.98	0.59	1.62	1.13	0.70	1.81	1.31	0.76	2.28	1.01	0.61	1.68	0.46*	0.26	0.83	0.82	0.51	1.31
Hispanic	0.97	0.58	1.63	0.81	0.52	1.27	1.02	0.63	1.65	0.94	0.58	1.50	1.10	0.59	2.06	1.14	0.70	1.83
Asian	0.83	0.50	1.39	0.68	0.41	1.11	0.91	0.53	1.54	0.94	0.54	1.62	1.54	0.70	3.38	1.10	0.66	1.83
Depression status by Race/ethnicity																		
Depressed Whites	Ref			Ref			Ref			Ref			Ref			Ref		
Depressed Black	0.71	0.14	3.52	1.06	0.20	5.63	2.51	0.43	14.79	0.95	0.19	4.67	3.65	0.32	41.07	1.25	0.28	5.48
Depressed Hispanic	2.05	0.39	10.79	3.34	0.79	14.10	5.42*	1.02	28.81	0.95	0.21	4.26	1.47	0.30	7.05	2.35	0.55	10.00
Depressed Asian	0.62	0.04	10.87	3.53	0.29	42.86	0.95	0.08	11.65	1.93	0.17	21.29	0.99	0.08	13.08	1.00^a^		

*Note*: *p < 0.05, **p < 0.01, ***p < 0.001. Models were adjusted for age at survey, race/ethnicity, marital status, educational level, Medicare plan, region, survey administration mode, survey year, time from diagnosis to survey completion, number of self‐reported comorbidities, tumor stage, receipt of surgery, and receipt of radiation therapy.

^a^ Insufficient variability.

## 4 DISCUSSION

Results of this study suggest that a recent history of clinical depression among breast cancer survivors is negatively associated with reports of patient experiences with care, specifically reports of their ability to get needed care, ratings of overall care, and ratings of care by a specialist. We found that about 8% of our sample met our criteria for clinical depression, which is comparable to a study that estimated the prevalence of clinical depression after breast cancer of about 6%,^30^ and another study which estimated the prevalence of depression after cancer using claims data at 9%.^31^ Our findings not only are consistent with previous research indicating that individuals with depressive symptoms report worse experiences with care compared to those without depressive symptoms,^17^ but they also augment previous published findings by examining the effects of clinically‐diagnosed depression, not symptomatology, on patient‐reported experiences with care among breast cancer survivors.

Ratings of health care experiences directly assess survivors' perceptions of the care they receive, and our results suggest that survivors with a recent history of depression reported having less than excellent encounters with the healthcare system. Their ratings might reflect survivors' perceptions of poor coordination of psychiatric and physical health needs. Having unmet care needs may negatively impact survivor's quality of life;^32^ therefore, future research should examine how reported experiences with care may affect getting survivorship‐related care among patients experiencing depression, and examine ways to address and improve overall quality of care in this population.

In our study population, survivors with a recent history of depression were more likely to be older, report more comorbidities, and complete the survey by mail. This could reflect an increased need to utilize more services than non‐depressed survivors, which could expose them to more challenges navigating the healthcare system, and experiencing more challenging encounters could explain, in part, their poorer ratings of experiences with care. However, our study did not examine use of health services or number of encounters; therefore, this assumption needs to be confirmed in future research. Future research also should examine mechanisms by which depression status influences experiences with care.

Our findings suggest that the association between depression and reports of the survivors' ability to get needed care differs for Hispanics compared to Whites. This finding is consistent with previous research results showing that Hispanics have trouble accessing care and report lower ratings in their ability to get care, compared to Whites.^33^ Future research should further examine pathways by which depression is linked to getting needed care among Hispanics, and identify strategies that might help link vulnerable patients to high‐quality care services, regardless of mental status or minority background. Our findings should be interpreted with caution because we had limited statistical power to detect differences in our stratified analysis. We did not find evidence to support other racial and ethnic differences in the influence of depression on ratings of care. The published literature on this topic is scant. Other investigators have found that Black non‐Hispanic women with depression report better ratings of provider communication, compared to White non‐Hispanic depressed women.^34^ However, their results were derived from a cancer‐free sample of the Medicare Expenditure Panel Survey (MEPS), where 82% of the respondents were younger than 65 years. Studies with a larger and more diverse sample should be conducted to further explore racial and ethnic differences.

### 4.1 Clinical implications

Survivors with a recent history of depression appear to be particularly vulnerable to difficulties in some of their healthcare encounters, as reflected by their reports of experiences with care. This highlights the need for additional supportive care services through Medicare plans among this subgroup of breast cancer survivors. Furthermore, findings from this study can help promote clinician training on interdisciplinary survivorship care, in hopes to improve health care experiences of breast cancer patients experiencing depression. Interventions could be designed to train clinicians to accurately diagnose depression in their clinical encounters with their patients, improve coordination of care by enhancing relationships between PCPs and oncologists, and promote strategies which prioritize patient‐centered care.

### 4.2 Study strengths and limitations

Findings from this study can help identify gaps for improved delivery of care among breast cancer survivors with psychiatric needs enrolled in Medicare and with similar characteristics to our sample. To our knowledge, ours is the first study to use the SEER‐CAHPSs linked dataset to determine the influence of clinical depression status on ratings of care, and examine whether differential effects of race/ethnicity exist in this relation. It is important to note some of our study limitations. First, our findings may not be generalizable to all Medicare beneficiaries because our sample includes only fee‐for‐service beneficiaries living in SEER regions who answered a CAHPS survey. However, this is one of the first population‐based studies to report that clinical depression is associated with perceptions of certain experiences with care and to describe the implications that this may have on quality of care for patients experiencing depression. Second, due to the limited racial/ethnic diversity in our sample, our study is under powered to definitively identify certain associations, such as racial and ethnic differences. Also, we reported *p*‐values un‐corrected for multiple comparisons, as recommended in the literature, we advise that our results be taken with caution and future studies are conducted to confirm our observed associations.^35^ Our sample did not include individuals with psychiatric diagnosis other than depression, and did not include patients who may have been institutionalized. These populations may have unique needs and should be studied separately. Lastly, our study did not assess depression management because our SEER‐CAHPS dataset did not contain information about prescription records or claims. Future studies should explore whether use of psychotropic medications impact survivors' perceptions of their experiences with care.

## 5 CONCLUSIONS

Breast cancer survivors with a recent history of clinical depression had lower odds of having excellent reports of care on three patient experience domains, compared to those without a recent history of depression. Our findings have important clinical implications and can help advance our understanding of subpopulations who may be at risk of getting substandard care. Especially because substandard care, assessed by poor ratings of experiences with care, has been associated with poor cancer outcomes such as, colorectal cancer mortality and non‐adherence to colorectal cancer treatment guidelines.^4^, ^5^ We found that having a clinical diagnosis of depression may contribute to differential ratings of experiences with care, and that the effect of depression on patient experiences may be different depending on ethnic background. Thus, more work is needed to explore pathways that can further explain this relation, and also determine whether clinical depression impacts breast cancer‐related outcomes.

## AUTHORS' CONTRIBUTIONS

The first draft of the manuscript was written by MA and all authors commented on previous versions of the manuscript. MA, AJF, TAP, SWV contributed to the study conception, analysis and interpretation of results. All authors read and approved the final manuscript.

## FUNDING INFORMATION

This study used the linked SEER‐CAHPS data resource. The interpretation and reporting of these data are the sole responsibility of the authors. The authors acknowledge the efforts of the National Cancer Institute; the Centers for Medicare & Medicaid Services; Information Management Services (IMS), Inc.; and the Surveillance, Epidemiology, and End Results (SEER) Program tumor registries in the creation of the SEER‐CAHPS data resource. Efforts by MA were supported by a pre‐doctoral fellowship at the University of Texas Health Science Center at Houston, School of Public Health, Susan G. Komen Traineeship in Breast Cancer Disparities (GTDR14300827). SEER‐CAHPS data were procured by AJF with support from a University of Texas Health Science Center at Houston School of Public Health Cancer Education and Career Development Program grant from the National Cancer Institute (R25‐CA57712). The contents of this manuscript are solely the responsibility of the authors and do not necessarily represent the official views of the funding bodies.

## ETHICS APPROVAL

This study was reviewed and granted exempt status by the UTHealth Committee for Protection of Human Subjects (HSC‐SPH‐20‐0812).

## Supporting information


Table A1

## Data Availability

The data that support the findings of this study are available from the corresponding author, MA, upon reasonable request.

## References

[1] Shaller D . Patient‐centered care: what does it take? . Commonwealth Fund; 2007.

[2] Kent, E. SEER‐CAHPS Overview. 2019; Available from: https://healthcaredelivery.cancer.gov/media/SEER‐CAHPS_Apr2019_WebinarSlides_Final_Final508.pdf

[3] Anhang Price R , Elliott MN , Zaslavsky AM , et al. Examining the role of patient experience surveys in measuring health care quality. Med Care Res Rev. 2014;71(5):522‐554. 25027409 10.1177/1077558714541480 PMC4349195

[4] Farias AJ , Ochoa CY , Toledo G , Bang SI , Hamilton AS , du XL . Racial/ethnic differences in patient experiences with health care in association with earlier stage at breast cancer diagnosis: findings from the SEER‐CAHPS data. Cancer Causes Control. 2020;31(1):13‐23. 31797123 10.1007/s10552-019-01254-3 PMC7443934

[5] Halpern MT , Urato MP , Lines LM , Cohen JB , Arora NK , Kent EE . Healthcare experience among older cancer survivors: analysis of the SEER‐CAHPS dataset. J Geriatr Oncol. 2018;9(3):194‐203. 29249645 10.1016/j.jgo.2017.11.005 PMC6002869

[6] Mollica MA , Enewold LR , Lines LM , et al. Examining colorectal cancer survivors' surveillance patterns and experiences of care: a SEER‐CAHPS study. Cancer Causes Control. 2017;28(10):1133‐1141. 28866818 10.1007/s10552-017-0947-2

[7] Mollica MA , Weaver KE , McNeel TS , Kent EE . Examining urban and rural differences in perceived timeliness of care among cancer patients: a SEER‐CAHPS study. Cancer. 2018;124(15):3257‐3265. 29878305 10.1002/cncr.31541

[8] Grassi L , Riba M . Cancer and severe mental illness: bi‐directional problems and potential solutions. Psychooncology. 2020;29(10):1445‐1451. 32915468 10.1002/pon.5534

[9] Carvalho AF , Hyphantis T , Sales PMG , et al. Major depressive disorder in breast cancer: a critical systematic review of pharmacological and psychotherapeutic clinical trials. Cancer Treat Rev. 2014;40(3):349‐355. 24084477 10.1016/j.ctrv.2013.09.009

[10] Simon GE , VonKorff M , Barlow W . Health care costs of primary care patients with recognized depression. Arch Gen Psychiatry. 1995;52(10):850‐856. 7575105 10.1001/archpsyc.1995.03950220060012

[11] Goodwin JS , Zhang DD , Ostir GV . Effect of depression on diagnosis, treatment, and survival of older women with breast cancer. J Am Geriatr Soc. 2004;52(1):106‐111. 14687323 10.1111/j.1532-5415.2004.52018.x PMC1853251

[12] Desai MM , Bruce ML , Kasl SV . The effects of major depression and phobia on stage at diagnosis of breast cancer. Int J Psychiatry Med. 1999;29(1):29‐45. 10376231 10.2190/0C63-U15V-5NUR-TVXE

[13] Zabora J , BrintzenhofeSzoc K , Curbow B , Hooker C , Piantadosi S . The prevalence of psychological distress by cancer site. Psycho‐oncology: journal of the psychological, social and behavioral dimensions of Cancer. 2001;10(1):19‐28. 10.1002/1099-1611(200101/02)10:1<19::aid-pon501>3.0.co;2-6 11180574

[14] Fann JR , Thomas‐Rich AM , Katon WJ , et al. Major depression after breast cancer: a review of epidemiology and treatment. Gen Hosp Psychiatry. 2008;30(2):112‐126. 18291293 10.1016/j.genhosppsych.2007.10.008

[15] Davidson J , Zisook S , Giller E , Helms M . Symptoms of interpersonal sensitivity in depression. Compr Psychiatry. 1989;30(5):357‐368. 2676337 10.1016/0010-440x(89)90001-1

[16] Martino SC , Elliott MN , Haviland AM , Saliba D , Burkhart Q , Kanouse DE . Comparing the health care experiences of Medicare beneficiaries with and without depressive symptoms in Medicare managed care versus fee‐for‐service. Health Serv Res. 2016;51(3):1002‐1020. 26368572 10.1111/1475-6773.12359 PMC4874814

[17] Martino SC , Elliott MN , Kanouse DE , Farley DO , Burkhart Q , Hays RD . Depression and the health care experiences of Medicare beneficiaries. Health Serv Res. 2011;46(6pt1):1883‐1904. 21762146 10.1111/j.1475-6773.2011.01293.x PMC3197881

[18] Mayberry RM , Nicewander DA , Qin H , Ballard DJ . Improving quality and reducing inequities: a challenge in achieving best care. Baylor University Medical Center Proceedings. Vol 19. Taylor & Francis; 2006:103‐118. 16609733 10.1080/08998280.2006.11928138 PMC1426185

[19] American Cancer Society . Breast Cancer Facts & Figures 2019–2020. American Cancer Society; 2019.

[20] Bailey ZD , Krieger N , Agénor M , Graves J , Linos N , Bassett MT . Structural racism and health inequities in the USA: evidence and interventions. Lancet. 2017;389(10077):1453‐1463. 28402827 10.1016/S0140-6736(17)30569-X

[21] Halpern MT , Urato MP , Kent EE . The health care experience of patients with cancer during the last year of life: analysis of the SEER‐CAHPS data set. Cancer. 2017;123(2):336‐344. 27654842 10.1002/cncr.30319

[22] Chawla N , Urato M , Ambs A , et al. Unveiling SEER‐CAHPS®: a new data resource for quality of care research. J Gen Intern Med. 2015;30(5):641‐650. 25586868 10.1007/s11606-014-3162-9 PMC4395616

[23] Center for Medicare advocacy . Medicare for people under 65. 2016. Available from: https://medicareadvocacy.org/under‐65‐project/.

[24] National Cancer Institute . CAHPS data documentation: SEER‐CAHPS details for researchers. 2020. Available from: https://healthcaredelivery.cancer.gov/seer‐cahps/aboutdata/documentation.html

[25] National Cancer Institute . Guidance on analytic approaches when modeling items and composites in analyses using SEER‐CAHPS data. 2019. Available from: https://healthcaredelivery.cancer.gov/seer‐cahps/researchers/approaches_guidance.html

[26] Sciences, N.C.I.D.o.c.c.p . SEER‐CAHPS data documentation. 2021. Available from: https://healthcaredelivery.cancer.gov/seer‐cahps/aboutdata/documentation.html

[27] Lines LM , Cohen J , Halpern MT , Smith AW , Kent EE . Care experiences among dually enrolled older adults with cancer: SEER‐CAHPS, 2005‐2013. Cancer Causes Control. 2019;30(10):1137‐1144. 31422490 10.1007/s10552-019-01218-7 PMC6786484

[28] National Cancer Institute . Handling Missing Data in SEER‐CAHPS. 2020. Available from: https://healthcaredelivery.cancer.gov/seer‐cahps/researchers/handling‐missing‐data.html

[29] StataCorp . Stata Statistical Software. StataCorp LP.; 2019.

[30] Danese MD , O'Malley C , Lindquist K , Gleeson M , Griffiths RI . An observational study of the prevalence and incidence of comorbid conditions in older women with breast cancer. Ann Oncol. 2012;23(7):1756‐1765. 22039090 10.1093/annonc/mdr486 PMC3387819

[31] McDermott, CL , Bansal A , Ramsey SD , Lyman GH , Sullivan SD , Depression and health care utilization at end of life among older adults with advanced non‐small‐cell lung cancer. J Pain Symptom Manage, 2018. 56(5): 699‐708.e1. 30121375 10.1016/j.jpainsymman.2018.08.004 PMC6226016

[32] Park BW , Hwang SY . Unmet needs and their relationship with quality of life among women with recurrent breast cancer. J Breast Cancer. 2012;15(4):454‐461. 23346176 10.4048/jbc.2012.15.4.454 PMC3542855

[33] Doty MM , Holmgren AL . Health care disconnect: gaps in coverage and care for minority adults. The Commonwealth Fund. 2006;21:1‐12. 16892521

[34] Keller AO , Gangnon R , Witt WP . Favorable ratings of providers' communication behaviors among U.S. women with depression: a population‐based study applying the behavioral model of health services use. Womens Health Issues. 2013;23(5):e309‐e317. 23993478 10.1016/j.whi.2013.07.002 PMC3828677

[35] Althouse AD . Adjust for multiple comparisons? It's not that simple. Ann Thorac Surg. 2016;101(5):1644‐1645. 27106412 10.1016/j.athoracsur.2015.11.024

