# Supplementary material for: Do breast cancer survivors with a recent history of clinical depression report worse experiences with care? A retrospective cohort study using SEER‐CAHPS data
Source: Cancer Med. 2022 Aug 5;12(2):1949–60. doi: 10.1002/cam4.5031 (PMC9883547; doi:10.1002/cam4.5031)
Supplement: Supplementary file 1 — Table A1 [file CAM4-12-1949-s001.docx]

# Supplemental Appendices

**Table A1**. Results from saturated logistic regression models assessing the effect of depression on reported experiences with care, adjusted for demographic and clinical characteristics

|  | **Doctor Communication** | | | **Getting Care Quickly** | | | | **Getting Needed Care** | | | | **Getting Prescription Drugs** | | | | **Care by Specialist** | | | **Overall Care** | | |
| --- | --- | --- | --- | --- | --- | --- | --- | --- | --- | --- | --- | --- | --- | --- | --- | --- | --- | --- | --- | --- | --- |
|  | OR | 95% | CI | | OR | 95% | CI | | OR | 95% | CI | | OR | 95% | CI | OR | 95% | CI | OR | 95% | CI |
| *N analyzed* | 1,698 |  |  | | 1,747 |  |  | | 1,660 |  |  | | 2,028 |  |  | 1,332 |  |  | 1,785 |  |  |
| ***Depression status*** |  |  |  | |  |  |  | |  |  |  | |  |  |  |  |  |  |  |  |  |
| *No* | Ref |  |  | | Ref |  |  | | Ref |  |  | | Ref |  |  | Ref |  |  | Ref |  |  |
| *Yes* | 0.89 | 0.60 | 1.32 | | 0.77 | 0.54 | 1.11 | | **0.58**** | **0.40** | **0.84** | | 0.74 | 0.50 | 1.08 | **0.49**** | **0.31** | **0.76** | **0.61**** | **0.42** | **0.89** |
| ***Age at survey*** | 1.00 | 0.98 | 1.01 | | 0.99 | 0.97 | 1.01 | | 1.02 | 1.00 | 1.03 | | 1.01 | 1.00 | 1.03 | 1.00 | 0.98 | 1.03 | 1.00 | 0.98 | 1.02 |
| ***Race/Ethnicity*** |  |  |  | |  |  |  | |  |  |  | |  |  |  |  |  |  |  |  |  |
| *Non-Hispanic White* | Ref |  |  | | Ref |  |  | | Ref |  |  | | Ref |  |  | Ref |  |  | Ref |  |  |
| *Non-Hispanic Black* | 0.95 | 0.59 | 1.53 | | 1.13 | 0.72 | 1.78 | | 1.44 | 0.85 | 2.44 | | 1.01 | 0.62 | 1.63 | **0.50*** | **0.28** | **0.89** | 0.84 | 0.53 | 1.32 |
| *Hispanic* | 1.05 | 0.64 | 1.72 | | 0.92 | 0.60 | 1.41 | | 1.21 | 0.76 | 1.93 | | 0.93 | 0.59 | 1.46 | 1.16 | 0.65 | 2.08 | 1.26 | 0.80 | 1.98 |
| *Asian* | 0.82 | 0.49 | 1.36 | | 0.71 | 0.44 | 1.16 | | 0.91 | 0.54 | 1.53 | | 0.97 | 0.57 | 1.66 | 1.54 | 0.73 | 3.27 | 1.04 | 0.63 | 1.71 |
| ***Marital Status*** |  |  |  | |  |  |  | |  |  |  | |  |  |  |  |  |  |  |  |  |
| *Not Married* | Ref |  |  | | Ref |  |  | | Ref |  |  | | Ref |  |  | Ref |  |  | Ref |  |  |
| *Married* | 0.96 | 0.77 | 1.20 | | 0.86 | 0.70 | 1.06 | | 1.10 | 0.88 | 1.37 | | 1.15 | 0.92 | 1.45 | 1.03 | 0.78 | 1.35 | 0.93 | 0.75 | 1.15 |
| *Missing* | 1.33 | 0.66 | 2.67 | | 0.62 | 0.33 | 1.16 | | 1.56 | 0.75 | 3.24 | | 1.38 | 0.67 | 2.85 | 1.06 | 0.45 | 2.46 | 0.87 | 0.47 | 1.61 |
| ***Education Level*** |  |  |  | |  |  |  | |  |  |  | |  |  |  |  |  |  |  |  |  |
| *HS or Less* | Ref |  |  | | Ref |  |  | | Ref |  |  | | Ref |  |  | Ref |  |  | Ref |  |  |
| *College+* | 0.81 | 0.65 | 1.01 | | **0.81*** | **0.66** | **1.00** | | **0.74**** | **0.59** | **0.92** | | **0.75*** | **0.60** | **0.94** | 0.83 | 0.63 | 1.09 | 0.96 | 0.78 | 1.18 |
| *Missing* | 1.27 | 0.59 | 2.74 | | 1.14 | 0.60 | 2.18 | | 0.75 | 0.37 | 1.50 | | 0.57 | 0.26 | 1.26 | 1.22 | 0.44 | 3.33 | 0.96 | 0.48 | 1.94 |
| ***Number of self-reported comorbidities*** |  |  |  | |  |  |  | |  |  |  | |  |  |  |  |  |  |  |  |  |
| *0* | Ref |  |  | | Ref |  |  | | Ref |  |  | | Ref |  |  | Ref |  |  | Ref |  |  |
| *1* | **0.73*** | **0.56** | **0.95** | | 0.87 | 0.68 | 1.11 | | **0.72*** | **0.56** | **0.93** | | 0.88 | 0.68 | 1.15 | **0.70*** | **0.50** | **0.97** | **0.69**** | **0.54** | **0.88** |
| *2+* | 0.91 | 0.63 | 1.31 | | **0.62**** | **0.45** | **0.85** | | 0.78 | 0.55 | 1.11 | | 0.81 | 0.57 | 1.14 | 0.78 | 0.51 | 1.19 | 0.78 | 0.56 | 1.10 |
| ***Tumor stage*** |  |  |  | |  |  |  | |  |  |  | |  |  |  |  |  |  |  |  |  |
| *I* | Ref |  |  | | Ref |  |  | | Ref |  |  | | Ref |  |  | Ref |  |  | Ref |  |  |
| *II -III* | 1.02 | 0.82 | 1.27 | | 0.84 | 0.68 | 1.02 | | 1.05 | 0.84 | 1.30 | | 0.95 | 0.76 | 1.19 | 0.94 | 0.72 | 1.23 | 0.99 | 0.80 | 1.22 |
| ***Medicare Status*** |  |  |  | |  |  |  | |  |  |  | |  |  |  |  |  |  |  |  |  |
| *FFS PDP* | Ref |  |  | | Ref |  |  | | Ref |  |  | | Ref |  |  | Ref |  |  | Ref |  |  |
| *FFS Only* | 1.05 | 0.75 | 1.48 | | 1.14 | 0.85 | 1.53 | | 1.24 | 0.90 | 1.70 | | **2.34***** | **1.76** | **3.10** | 0.77 | 0.52 | 1.16 | 1.27 | 0.93 | 1.73 |
| ***Surgery*** |  |  |  | |  |  |  | |  |  |  | |  |  |  |  |  |  |  |  |  |
| *No* | Ref |  |  | | Ref |  |  | | Ref |  |  | | Ref |  |  | Ref |  |  | Ref |  |  |
| *Yes* | 1.12 | 0.27 | 4.66 | | 0.37 | 0.08 | 1.77 | | 0.53 | 0.09 | 3.03 | | 0.36 | 0.04 | 3.05 | 0.41 | 0.05 | 3.59 | 0.43 | 0.09 | 2.09 |
| *Missing* | 1.06 | 0.15 | 7.46 | | 0.20 | 0.03 | 1.47 | | 0.26 | 0.03 | 2.35 | | 0.45 | 0.03 | 6.28 | 0.44 | 0.03 | 6.83 | 0.44 | 0.06 | 3.27 |
| ***Radiation treatment*** |  |  |  | |  |  |  | |  |  |  | |  |  |  |  |  |  |  |  |  |
| *No* | Ref |  |  | | Ref |  |  | | Ref |  |  | | Ref |  |  | Ref |  |  | Ref |  |  |
| *Yes* | 1.04 | 0.84 | 1.30 | | 0.99 | 0.81 | 1.20 | | 0.96 | 0.77 | 1.19 | | 0.95 | 0.76 | 1.18 | 1.05 | 0.80 | 1.37 | 1.01 | 0.82 | 1.24 |
| *Missing* | 0.87 | 0.28 | 2.66 | | 2.72 | 0.72 | 10.24 | | 1.30 | 0.32 | 5.19 | | 1.40 | 0.38 | 5.13 | 1.04 | 0.21 | 5.17 | 1.53 | 0.40 | 5.82 |
| ***Region*** |  |  |  | |  |  |  | |  |  |  | |  |  |  |  |  |  |  |  |  |
| *West* | Ref |  |  | | Ref |  |  | | Ref |  |  | | Ref |  |  | Ref |  |  | Ref |  |  |
| *Midwest* | **0.71** | **0.51** | **0.99** | | 0.86 | 0.63 | 1.18 | | 1.05 | 0.75 | 1.48 | | 0.80 | 0.57 | 1.12 | 1.29 | 0.82 | 2.04 | 1.00 | 0.72 | 1.39 |
| *Northeast* | 0.98 | 0.73 | 1.33 | | 0.98 | 0.75 | 1.28 | | 0.97 | 0.72 | 1.29 | | 1.07 | 0.79 | 1.46 | 0.86 | 0.61 | 1.21 | 0.93 | 0.70 | 1.22 |
| *South* | 0.91 | 0.68 | 1.23 | | 0.96 | 0.73 | 1.26 | | 1.23 | 0.91 | 1.66 | | 1.09 | 0.80 | 1.49 | 1.39 | 0.95 | 2.05 | 1.07 | 0.80 | 1.42 |
| ***Survey Administration Mode*** |  |  |  | |  |  |  | |  |  |  | |  |  |  |  |  |  |  |  |  |
| *Mail* | Ref |  |  | | Ref |  |  | | Ref |  |  | | Ref |  |  | Ref |  |  | Ref |  |  |
| *Phone* | 1.03 | 0.76 | 1.41 | | 1.23 | 0.93 | 1.64 | | 0.76 | 0.55 | 1.06 | | 1.15 | 0.85 | 1.55 | 0.83 | 0.54 | 1.27 | 1.04 | 0.77 | 1.40 |
| ***Survey Year*** |  |  |  | |  |  |  | |  |  |  | |  |  |  |  |  |  |  |  |  |
| *2000-2004* | Ref |  |  | | Ref |  |  | | Ref |  |  | | Ref |  |  | Ref |  |  | Ref |  |  |
| *2007-2010* | **2.15***** | **1.44** | **3.21** | | 1.08 | 0.74 | 1.59 | | **0.43***** | **0.28** | **0.66** | | 0.90 | 0.57 | 1.43 | 1.13 | 0.70 | 1.83 | 0.86 | 0.58 | 1.29 |
| *2011-2013* | **2.25***** | **1.57** | **3.23** | | 1.24 | 0.87 | 1.77 | | **0.49***** | **0.33** | **0.73** | | 1.14 | 0.74 | 1.77 | 1.37 | 0.88 | 2.13 | 0.94 | 0.65 | 1.36 |
| ***Time from diagnosis to survey completion*** | 1.00 | 1.00 | 1.00 | | 1.00 | 1.00 | 1.00 | | **1.00***** | **0.99** | **1.00** | | 1.00 | 0.99 | 1.00 | 1.00 | 0.99 | 1.00 | 1.00 | 1.00 | 1.00 |

*p < .05, **p < .01, *** p < .001;

**Table A2**. Results from stratified analysis - assessing the effect of depression on getting needed care, adjusted for demographic and clinical characteristics among HISPANICS

|  | Getting Needed Care | | |
| --- | --- | --- | --- |
|  | **OR** | **95%** | **CI** |
| *N analyzed* | 95 |  |  |
| *Depression status* |  |  |  |
| *No* | Ref |  |  |
| *Yes* | 1.80 | 0.32 | 10.19 |
| *Age at survey* | 0.99 | 0.91 | 1.07 |
| *Marital Status* |  |  |  |
| *Not Married* | Ref |  |  |
| *Married* | 2.7 | 0.92 | 7.88 |
| *Missing* | 1.00• |  |  |
| *Education Level* |  |  |  |
| *HS or less* | Ref |  |  |
| *College+* | 0.49 | 0.16 | 1.50 |
| *Missing* | 0.17 | 0.01 | 1.47 |
| *Number of self-reported comorbidities* |  |  |  |
| *0* | Ref |  |  |
| *1* | 0.88 | 0.27 | 2.79 |
| *2+* | 5.52 | 0.90 | 34.11 |
| *Tumor stage* |  |  |  |
| *I* | Ref |  |  |
| *II -III* | 0.93 | 0.33 | 2.60 |
| *Medicare Status* |  |  |  |
| *FFS PDP* | Ref |  |  |
| *FFS Only* | 0.42 | 0.07 | 2.24 |
| *Surgery* |  |  |  |
| *No* | 1.00• |  |  |
| *Yes* | 1.00• |  |  |
| *Radiation treatment* |  |  |  |
| *No* | Ref |  |  |
| *Yes* | 0.94 | 0.31 | 2.82 |
| *Region* |  |  |  |
| *West* | Ref |  |  |
| *Midwest* | 1.00 |  |  |
| *Northeast* | 0.87 | 0.22 | 3.37 |
| *South* | 0.94 | 0.14 | 6.45 |
| *Survey Administration Mode* |  |  |  |
| *Mail* | Ref |  |  |
| *Phone* | 0.79 | 0.20 | 2.96 |
| *Survey Year* |  |  |  |
| *2000-2004* | Ref |  |  |
| *2007-2010* | 0.18 | 0.02 | 1.59 |
| *2011-2013* | 0.33 | 0.53 | 2.10 |
| *Time from diagnosis to survey completion* | 1.00 | 0.98 | 1.01 |

Notes:

*p < .05, **p < .01, *** p < .001

• not estimated due to low cell count on these categories
